# “Dry Tap” Fine-Needle Aspiration Biopsy as a Diagnostic Clue in Cyst-like Juvenile Jaw Lesions Mimicking Dentigerous Cysts on Panoramic Radiography and Cone-Beam Computed Tomography

**DOI:** 10.3390/diagnostics16030439

**Published:** 2026-02-01

**Authors:** Kamil Nelke, Klaudiusz Łuczak, Ömer Uranbey, Büşra Ekinci, Angela Rosa Caso, Michał Gontarz, Maciej Janeczek, Zygmunt Stopa, Piotr Kuropka, Maciej Dobrzyński

**Affiliations:** 1 Maxillo-Facial Surgery Ward, EMC Hospital, Pilczycka 144, 54-144 Wrocław, Poland; 2 Academy of Applied Sciences, Health Department, Academy of Silesius in Wałbrzych, Zamkowa 4, 58-300 Wałbrzych, Poland; 3 Department of Oral and Maxillofacial Surgery, Faculty of Dentistry, Aydın Adnan Menderes University, 09010 Aydın, Türkiye; 4 Department of Medical Pathology, Faculty of Medicine, Aydın Adnan Menderes University, 09010 Aydın, Türkiye; 5 Department of Oral and Maxillo-Facial Surgery, University of Siena, Viale Aldo Moro, 2, 53100 Siena, SI, Italy; 6 Department of Cranio-Maxillofacial Surgery, Jagiellonian University Medical College, Jakubowskiego 2 Street, 30-688 Cracow, Poland; michal.gontarz@uj.edu.pl; 7 Department of Biostructure and Animal Physiology, Wrocław University of Environmental and Life Sciences, Kożuchowska 1, 51-631 Wrocław, Poland; 8 Department of Cranio-Maxillofacial Surgery, Oral Surgery and Implantology, Medical University of Warsaw, Lindleya 4, 02-005 Warsaw, Poland; 9 Division of Histology and Embryology, Department of Biostructure and Animal Physiology, Wrocław University of Environmental and Life Sciences, Cypriana K. Norwida 25, 50-375 Wrocław, Poland; 10 Department of Pediatric Dentistry and Preclinical Dentistry, Wrocław Medical University, Krakowska 26, 50-425 Wrocław, Poland

**Keywords:** juvenile jaw, maxillary bone, cysts and tumors, ameloblastic fibroma, rhinosinusitis, case report

## Abstract

Pediatric odontogenic tumors are rare but are frequently overlooked because they often mimic simple cysts on routine radiographic examinations. The radiographic appearance on panoramic imaging and cone-beam computed tomography (CBCT) frequently does not correlate with the true biological nature of these lesions. On CBCT, classic odontogenic tumors often demonstrate mixed radiolucent–radiopaque patterns with ill-defined borders, internal calcifications, septations, or other structural features. The diagnostic challenge arises when an odontogenic tumor mimics a unilateral, well-defined radiolucent area or a cystic lesion with clear borders and no associated tooth displacement, erosion, root resorption, or cortical bone dehiscence. Panoramic radiography has inherent diagnostic limitations but remains widely used for routine dental screening. CBCT provides enhanced three-dimensional assessment and improves diagnostic accuracy in the evaluation of jaw lesions. A marked increase in dental follicle diameter necessitates differentiation between cystic transformation, inflammatory processes, and other odontogenic pathologies. Cortical swelling and bone asymmetry warrant careful evaluation. In this context, an atypical cyst-like lesion detected on routine panoramic radiography prompted a needle aspiration biopsy, which revealed a dry tap and suggested a solid lesion. This prompted CBCT evaluation. Two juvenile cases are presented in which clinical findings, panoramic radiography, and CBCT provided discordant diagnostic impressions of cystic-appearing lesions with well-defined borders and bone expansion. These cases illustrate a diagnostic pathway in which imaging demonstrates a cyst-like appearance with benign radiological features, fine-needle aspiration biopsy reveals the absence of cystic fluid, and histopathology confirms that radiology alone cannot reliably distinguish true cysts from solid odontogenic tumors in pediatric patients.

**Figure 1:**
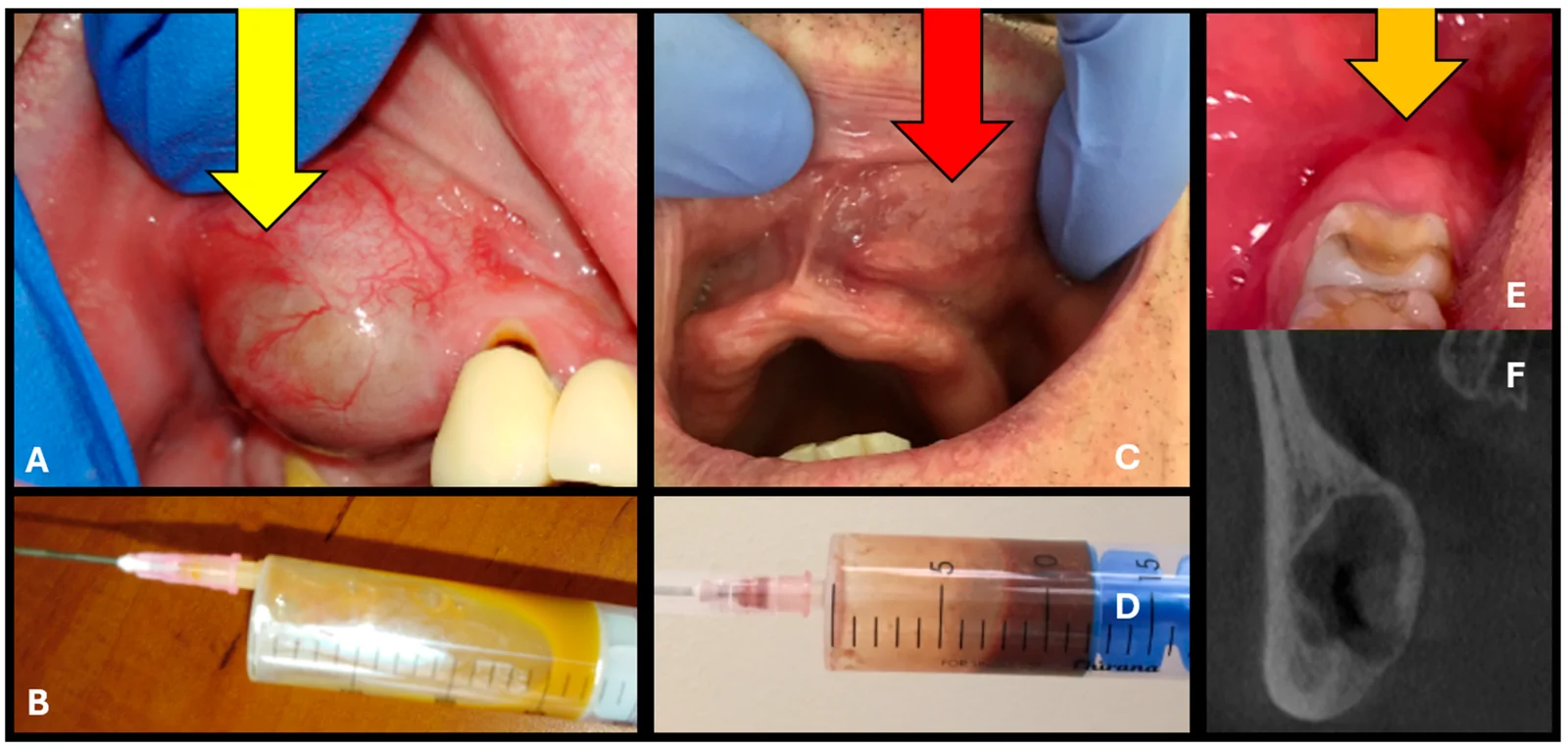
The oral cavity may harbor a broad spectrum of cysts, tumors, and other lesions. Cystic lesions may demonstrate fluctuation, an “eggshell cracking” sensation on palpation, and a well-circumscribed, rounded contour when the cortical bone is preserved. Other lesions may present as firm, solid masses without crepitus or fluctuation, with well-defined corticated borders, or show extracortical spread typical of advanced jaw pathology. While CBCT-based clinical evaluation, dental assessment, and histopathological analysis after biopsy offer high diagnostic accuracy, adjunctive rapid methods may further support lesion characterization. Fine-needle aspiration biopsy (FNAB) is a simple, minimally invasive procedure that involves inserting a needle into the lesion to obtain tissue or cellular material for diagnostic purposes. This method, although not definitive, represents a useful and often underutilized adjunctive diagnostic tool for evaluating lesion consistency during needle aspiration (NA). It allows reliable differentiation between solid and fluid-containing lesions. Furthermore, gathered tissue samples can be easily used for cytology and for initial evaluation [1,2,3,4]. Prarthana et al.’s study indicated that the concordance between cytological and histopathological diagnosis was 60% during needle aspiration biopsy [1]. On the other hand, Kumari et al. estimated that the sensitivity of FNAC in diagnosing bone lesions was 80% and the specificity was 88% [4]. In such cases, needle aspiration (NA) serves to assess the consistency and internal structure of jaw lesions, particularly in atypical presentations and in pediatric or elderly patients. Typical cystic lesions have well-defined borders on examination ((**A**), yellow arrow showing the mass). Following needle aspiration, a characteristic cholesterol-rich aspirate is usually obtained (**B**). In contrast, certain cyst-like tumors may closely mimic true cystic lesions on clinical and radiological assessment ((**C**), red arrow); however, needle aspiration in such cases may yield blood or atypical fluid rather than cystic content ((**D**)—case of UA, unicystic ameloblastoma). It should be noted that odontogenic cysts may occasionally be inflamed, in which case purulent material can be obtained on aspiration. In such situations, both cytological and microbiological analyses may provide additional diagnostic information. Lesions with irregular borders lacking calcifications or other solid tumor characteristics on imaging, particularly when closely associated with adjacent teeth, are more suggestive of odontogenic cysts. (**E**,**F**). When needle aspiration was performed ((**E**), orange arrow), a dry tap was obtained, and the final histopathological examination following excisional biopsy with ostectomy confirmed an ossifying fibroma of the mandible (OsF; images (**E**,**F**)). Needle aspiration biopsy remains underutilized in routine clinical practice, despite its potential value as an adjunctive diagnostic tool, especially in pediatric patients.

**Figure 2:**
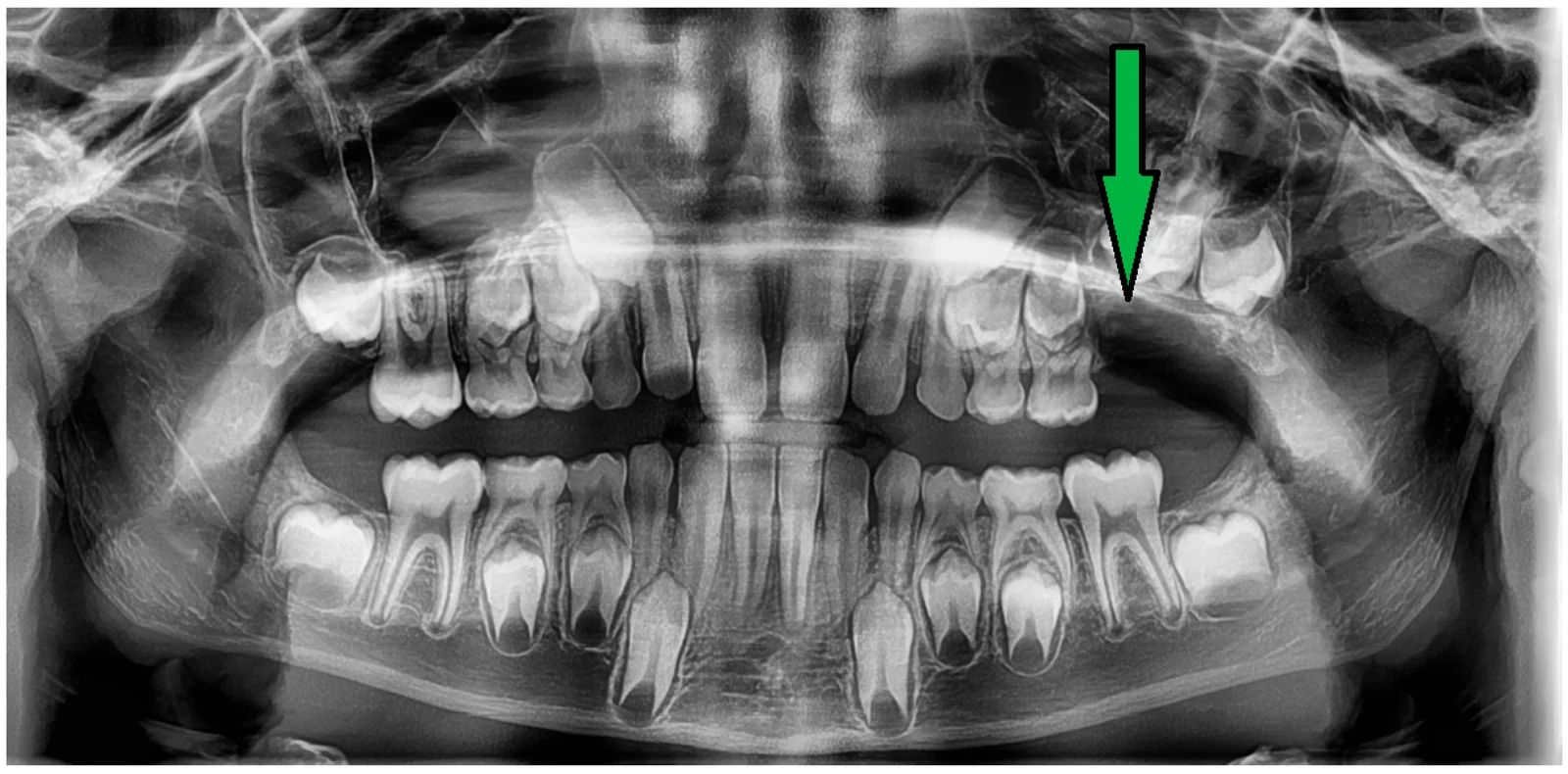
In juvenile patients, panoramic radiography is commonly used for screening, particularly during mixed dentition in orthodontic assessment and, in selected cases, otolaryngological evaluation. However, this developmental period is radiographically complex: multiple erupting teeth, enlarged follicles, overlapping roots, and asymptomatic lesions can easily obscure underlying pathology. Because odontogenic tumors are uncommon in children, they are often misinterpreted as benign cysts. Their silent development among erupting and impacted teeth further increases the risk of being overlooked. Detected lesions may present with variable local characteristics, including differences in morphology, extracortical extension, tooth displacement or resorption, bone swelling, and other associated features. In growing patients with mixed dentition and multiple developing tooth buds, careful assessment of the number, position, and eruption status of teeth remains essential for accurate interpretation. The timing of tooth eruption is well described in the literature; however, various local and systemic factors may lead to delayed tooth eruption, particularly those associated with follicular or eruption cysts. Moreover, certain odontogenic tumors can radiographically mimic cystic lesions, especially follicular cysts in juvenile patients, posing a significant diagnostic challenge. Although panoramic imaging can identify enlarged follicles or unilocular radiolucencies, its inherent limitations may obscure the true lesion size, cortical involvement, or internal architecture. On conventional panoramic radiographs, many small or atypical lesions within the jawbones, tooth buds, and tooth-bearing structures may be easily overlooked during routine screening. Enlarged, inflamed, or morphologically atypical eruption cysts (ECs), follicular cysts (FCs), or enlarged and inflamed dental follicles are frequently encountered on panoramic radiographs [5,6]. However, limitations in panoramic imaging interpretation may lead to underestimation or mischaracterization of lesion extent and features. Lesions associated with clinical bone swelling, expansion, or asymmetry require careful evaluation, as routine panoramic radiography has inherent limitations. In such cases, cone-beam computed tomography (CBCT) is commonly used to assess lesion extent and to distinguish among cystic, tumorous, and other osseous pathologies. Conventional computed tomography (CT) does not always allow detailed evaluation of lesions located between developing tooth buds and adjacent teeth. However, in advanced cases, CT is useful for assessing odontogenic tumor dimensions, volume, extracortical extension, soft tissue involvement, and lesion margins. A follicular cyst (FC) typically develops around the crown of an unerupted tooth, whereas an eruption cyst (EC) presents as a superficial, fluid-filled lesion arising in the soft tissues overlying an erupting tooth. Some odontogenic tumors may mimic either of the mentioned cysts, and cystic lesions include adenomatoid odontogenic tumor (AOT), unicystic ameloblastoma (UA), calcifying cystic odontogenic tumors, odontogenic fibroma (OF), ameloblastic fibroma (AF), clear cell odontogenic tumor/carcinoma, and rarely others [5,6,7]. Ameloblastic fibroma (AF) is a rare odontogenic tumor, representing approximately 2% of cases, that is slow-growing and generally self-limiting in the jawbones. It is commonly identified incidentally on radiographs as a cystic-appearing lesion associated with tooth retention or clinically as a solid, asymmetrical lesion. It typically presents as a well-circumscribed unilocular radiolucency with clear borders and a cyst-like appearance (Green arrow—radiolucent area in the left maxillary bone). In most cases, a single-step surgical approach involving excisional biopsy, bone curettage, and enucleation is sufficient, with repeat surgery required only rarely in larger lesions [8,9,10,11,12].

**Figure 3:**
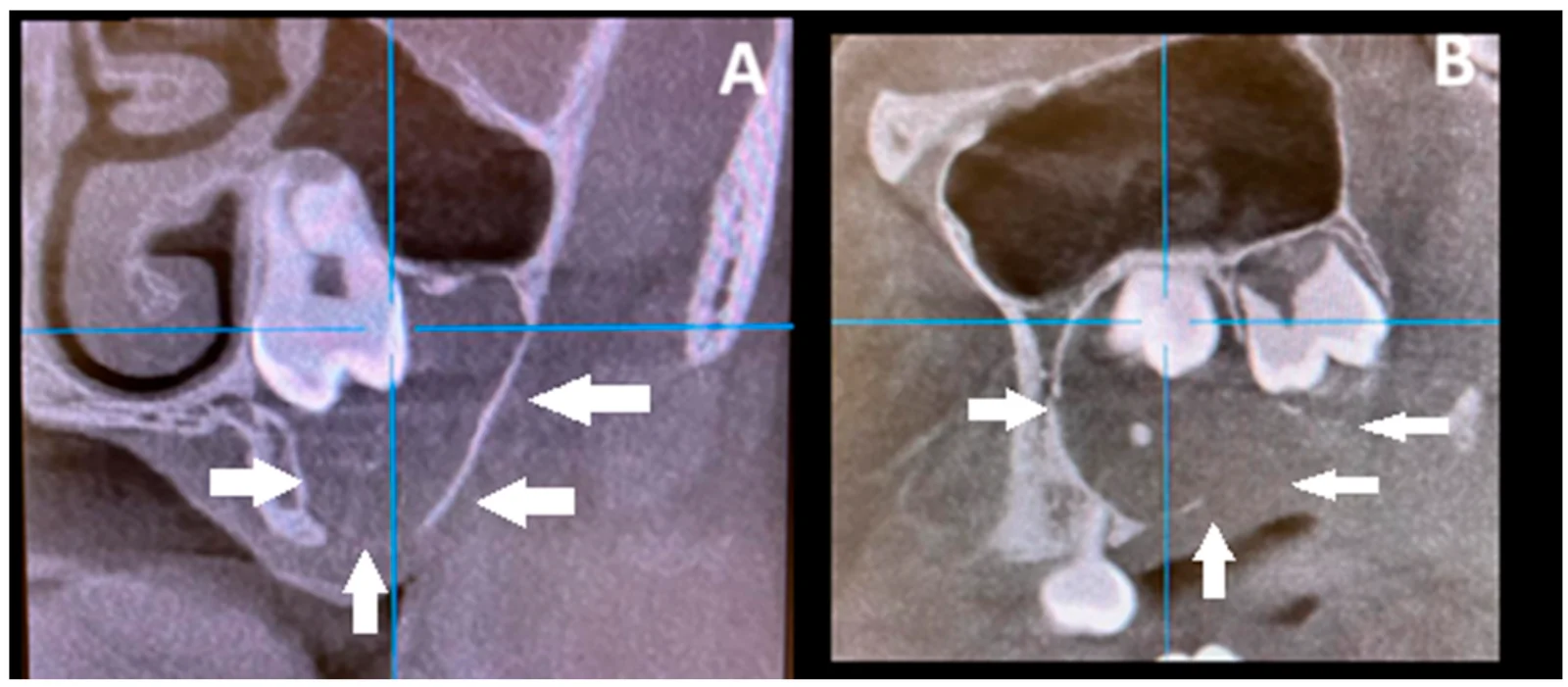
A nine-year-old patient showed no visible lesion on routine panoramic radiography; however, subtle bone asymmetry and suspicion of a solid structure on initial clinical evaluation, together with a history of rhinosinusitis, prompted CBCT for further diagnostic assessment. (**A**) Coronal view demonstrating an atypical unilocular, well-circumscribed radiolucency surrounding a totally impacted molar (white arrows indicating lesion borders), with mild bucco-palatal cortical expansion and facial asymmetry. (**B**) Sagittal view confirming the lesion’s cyst-like configuration and relationship to the impacted tooth. Coronal and sagittal views (vertical and horizontal blue lines point out a totally impacted and retained molar tooth in the left maxillary bone and the appearance of the lesion) show an atypical unicystic-like lesion and bone asymmetry with cortical expansion (white arrows the borders of radiolucent area). In certain cases, odontogenic lesions may be associated with rhinosinusitis, leading to referral for otolaryngological consultation [9,10]. Unilocular, cyst-like lesions in the oral cavity, particularly those associated with impacted or unerupted teeth, may mimic other odontogenic lesions, especially odontogenic tumors. A typical cyst is characterized by a fluid-filled cavity, whereas solid lesions lack an internal fluid component. The most common solid odontogenic tumors encountered in the jawbones of pediatric patients include ameloblastoma, calcifying cystic odontogenic tumor (CCOT), and, more rarely, the solid variant of odontogenic keratocyst (OKC), which has true destructive potential. These lesions may be misdiagnosed as unilateral, round, or oval radiolucent areas resembling unicystic ameloblastoma (UA) or OKC. A mixed radiolucent–radiopaque appearance, or the presence of a honeycomb or soap-bubble pattern on CBCT and panoramic imaging, may provide further insight into the underlying lesion type. The presence of bone swelling, cortical expansion, tooth resorption, or extracortical spread raises suspicion for more aggressive pathology. Solid, non-cystic growth patterns can be recognized through integrated radiological, clinical, and histopathological assessment, although true odontogenic tumors remain a clinical and surgical challenge in pediatric patients [7,8,9]. In the presented case, the presence of mild bone swelling and an impacted molar raised suspicion for an odontogenic tumor. Although CBCT suggested a cyst-like unilocular lesion, FNAB performed under local anesthesia yielded a dry tap, indicating a solid lesion consistent with an odontogenic tumor. All procedures were performed by oral and maxillofacial surgeons, imaging was interpreted by clinicians trained in dentomaxillofacial radiology, and histopathological assessment was performed by specialist pathologists. This highlights a key principle: when FNAB fails to demonstrate cystic fluid, the lesion should be approached as a tumor until proven otherwise. Nevertheless, some odontogenic tumors may mimic follicular cysts, especially when associated with unerupted permanent teeth, as reported by Petroni et al. [8,9,10]. Accordingly, an initial diagnosis of ameloblastic fibroma of the left maxilla mimicking an FC was made. This finding was supported by the solid-like appearance, absence of fluid, and mild bone swelling with expansile character of the lesion, but without any signs of tooth or bone erosion. The ameloblastic fibroma (AF) is a mixed benign odontogenic tumor that may have variants differentiated under microscopic evaluation [7,8]. Quite often, conventional AF includes solid/multicystic variants with both neoplastic epithelial and mesenchymal components. These variants include ameloblastic fibrodentinoma (AFD), ameloblastic fibro-odontoma (AFO), and the rare extraosseous peripheral ameloblastic fibroma. These entities can be differentiated based on the presence and degree of dental hard tissue formation. Moles et al. reported that most ameloblastic fibromas are associated with impacted or developing teeth [9,10,11,12,13,14]. Conversely, de Oliveira et al. demonstrated the diagnostic value of immunohistochemical expression of BRAF p.V600E and SOX9 in the clinicopathological differentiation of these lesions [10,11,12,13,14]. In the present case, given the well-defined lesion borders, absence of invasion, and lack of fluid on needle aspiration, excisional biopsy with curettage and ostectomy was performed under general anesthesia.

**Figure 4:**
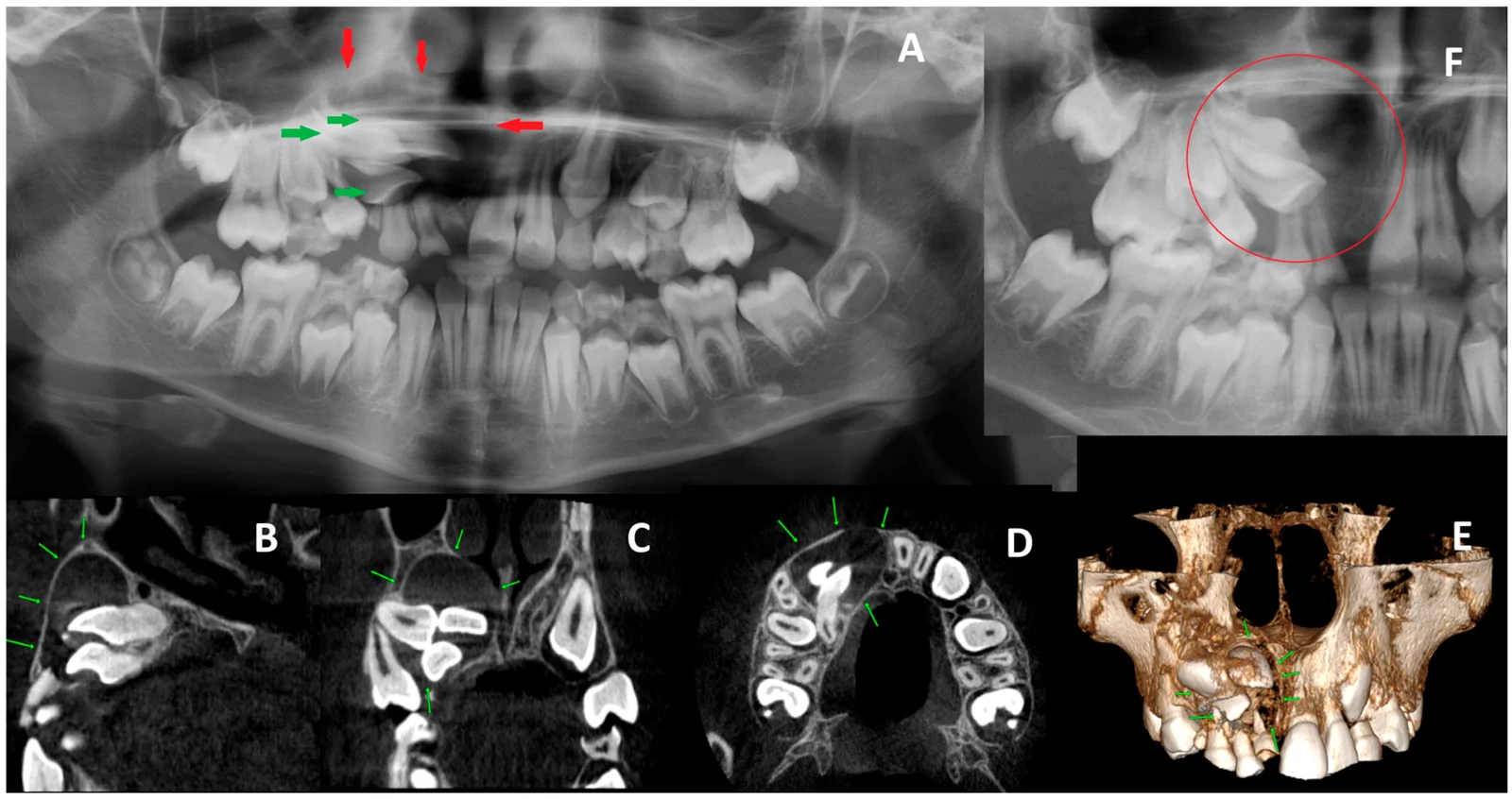
It is quite important to visualize other possible cystic and cystic-like lesions, which commonly present with well-defined borders and manifest as a radiolucent area in radiological studies. Most typically, FC is associated with the most impacted and retained teeth. Panoramic radiography remains the most common screening tool in pediatric oral and maxillofacial diagnostics; however, its two-dimensional projection limits accurate assessment of juvenile odontogenic lesions. To demonstrate how different odontogenic tumors may produce nearly identical radiographic appearances, we include a second case, a 9-year-old girl with asymmetric maxillary incisor eruption that also mimicked an FC on imaging but proved to be an odontogenic fibroma (OF). The panoramic radiograph ((**A**), with red arrows indicating the lesion borders and green arrows indicating the displaced unerupted teeth) showed an enlarged follicular radiolucency; however, the true extension, its relationship to adjacent developing teeth, and potential cortical involvement were not clearly visualized. CBCT imaging ((**B**–**E**), green arrows indicating the lesion borders) demonstrated that the lateral incisor and three canine tooth buds were entirely encased within a large unilocular structure occupying the anterior maxilla, revealing significant expansion that had been underestimated on OPG. FNAB yielded minimal or no fluid, which raised a strong suspicion of a solid odontogenic tumor rather than a simple cystic lesion. This finding was particularly relevant, as CBCT alone could not definitively differentiate between a follicular cyst and a unicystic odontogenic tumor. Histopathological examination confirmed the diagnosis of OF, characterized by a mature fibrous stroma with inactive odontogenic epithelial rests. Clinically, the primary incisors exhibited caries and mobility. Under general anesthesia, the lesion was enucleated, and the impacted permanent central incisor was extracted due to inadequate alveolar support, while the permanent lateral incisor and canine tooth buds were intentionally preserved to allow future orthodontic eruption. The surgical cavity demonstrated early osseous fill, consistent with the initial phase of reparative bone healing. At the six-month follow-up (**F**), no recurrence of the cystic lesion was detected, and the developing permanent teeth were observed to be anchored within the newly formed residual bone (red circle outlines the postoperative cystic cavity) [14]. Progressive bone regeneration within the excised cystic cavity was evident, and the patient was subsequently referred for orthodontic assessment to guide the eruption of the preserved teeth. Large OFs and follicular cysts may radiographically mimic unicystic ameloblastoma, ameloblastic fibroma, or even ameloblastic fibro-odontoma when significant tooth-bud displacement or bucco-palatal expansion occurs, leading to considerable diagnostic ambiguity and potential delays in treatment decisions [15,16]. The restricted spatial resolution of panoramic imaging further hampers differentiation between cystic and neoplastic pathologies in the developing maxilla [17,18]. By contrast, cone-beam computed tomography (CBCT) enables more precise visualization of lesion margins, internal septations, and cortical involvement, providing a superior depiction of three-dimensional morphology [15,16,17,18,19,20]. Both cases illustrate how CBCT improves visualization but still fails to differentiate cystic from solid odontogenic tumors when the radiographic pattern is deceptively benign. These findings collectively reinforce the role of CBCT as an indispensable adjunct to panoramic radiography in the diagnostic workflow of juvenile odontogenic tumors [21,22,23], although considerations regarding radiation exposure and accessibility remain important limitations. Beyond lesion identification, CBCT and histopathologic correlation offer valuable clues for differential diagnosis among fibrogenic and myxoid odontogenic tumors. When no epithelial remnants are detected histologically, and the lesion perforates the cortex to invade adjacent soft tissue while displaying thick, irregular internal septa, a more aggressive desmoplastic fibroma should be considered. In contrast, lesions with straight, delicate septa and a homogeneous myxoid matrix are more typical of odontogenic myxoma, whereas those with granular or coarse septa may suggest central giant cell granuloma, particularly when multinucleated giant cells are observed microscopically. Recognizing these imaging–histologic patterns greatly refines diagnostic precision and guides appropriate surgical planning. In this second case, the key challenge was not the recognition of a jaw lesion itself, but the false reassurance created by a classic follicular cyst-like pattern on OPG and an apparently benign, unilocular configuration on CBCT, despite the lesion’s true extent and the degree of tooth-bud encasement. Importantly, FNAB yielded minimal/no fluid, which was inconsistent with a true cystic cavity and immediately raised suspicion for a solid odontogenic process. The take-home message from both cases is, therefore, workflow-based: when a juvenile unilocular radiolucency looks ‘cystic’ on imaging, but aspiration does not produce cystic content, the lesion should be managed as potentially tumorous until definitive histopathology is obtained. This imaging aspiration discordance is the pivotal educational point, as it can prevent delayed diagnosis and inappropriate “cyst-first” assumptions in children.

**Figure 5:**
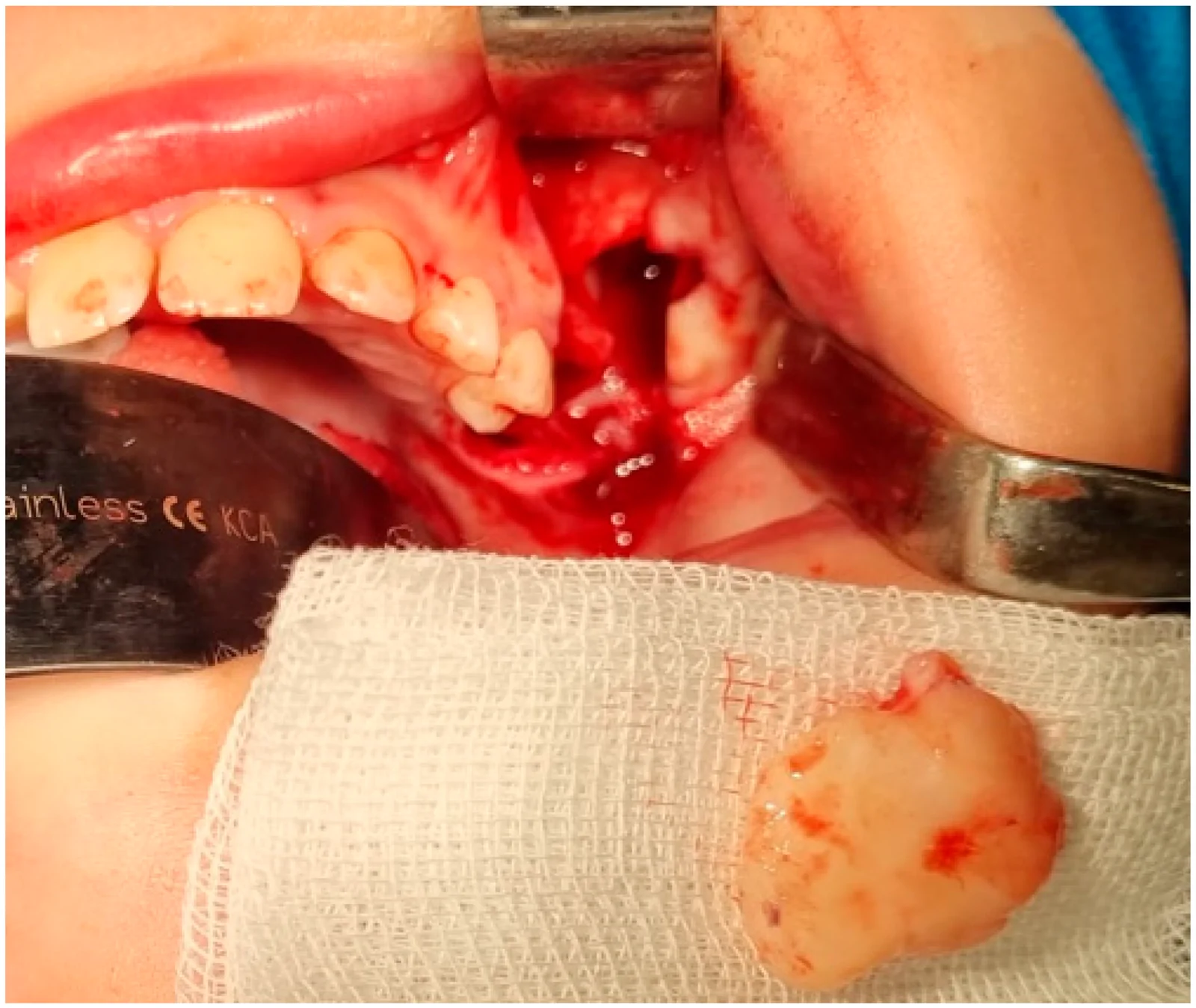
Under general anesthesia with orotracheal intubation, a standard trapezoidal flap was elevated. Given the solid nature of the lesion, the initial surgical step involved identifying the lesion margins relative to adjacent healthy bone, as well as the surrounding teeth and tooth buds. After surgical curettage, the lesion was removed en bloc without complications, as shown in Figure 5. Given its firm and solid consistency, additional curettage of the surrounding alveolar bone was performed to achieve local clearance while preserving adjacent teeth and tooth buds. In areas with exposed bone, a high-speed burr was used to perform additional ostectomy. All teeth and tooth buds were preserved, and resorbable collagen sponges were placed within the defect to support healing and facilitate the eruption of impacted and displaced teeth. Resorbable 4-0 PGA sutures were applied. The postoperative course was uneventful. Sutures were removed on postoperative day 10, and the patient was scheduled for further orthodontic and maxillofacial follow-up. Quite commonly, peak occurrence of AF is around 11–16 years of age, where 77% occur before 20 years of age, whereas OF is more common around 20–40 years of age, and its early occurrence is not common [1,2,3,4,5,6,7,8,9,10].

**Figure 6:**
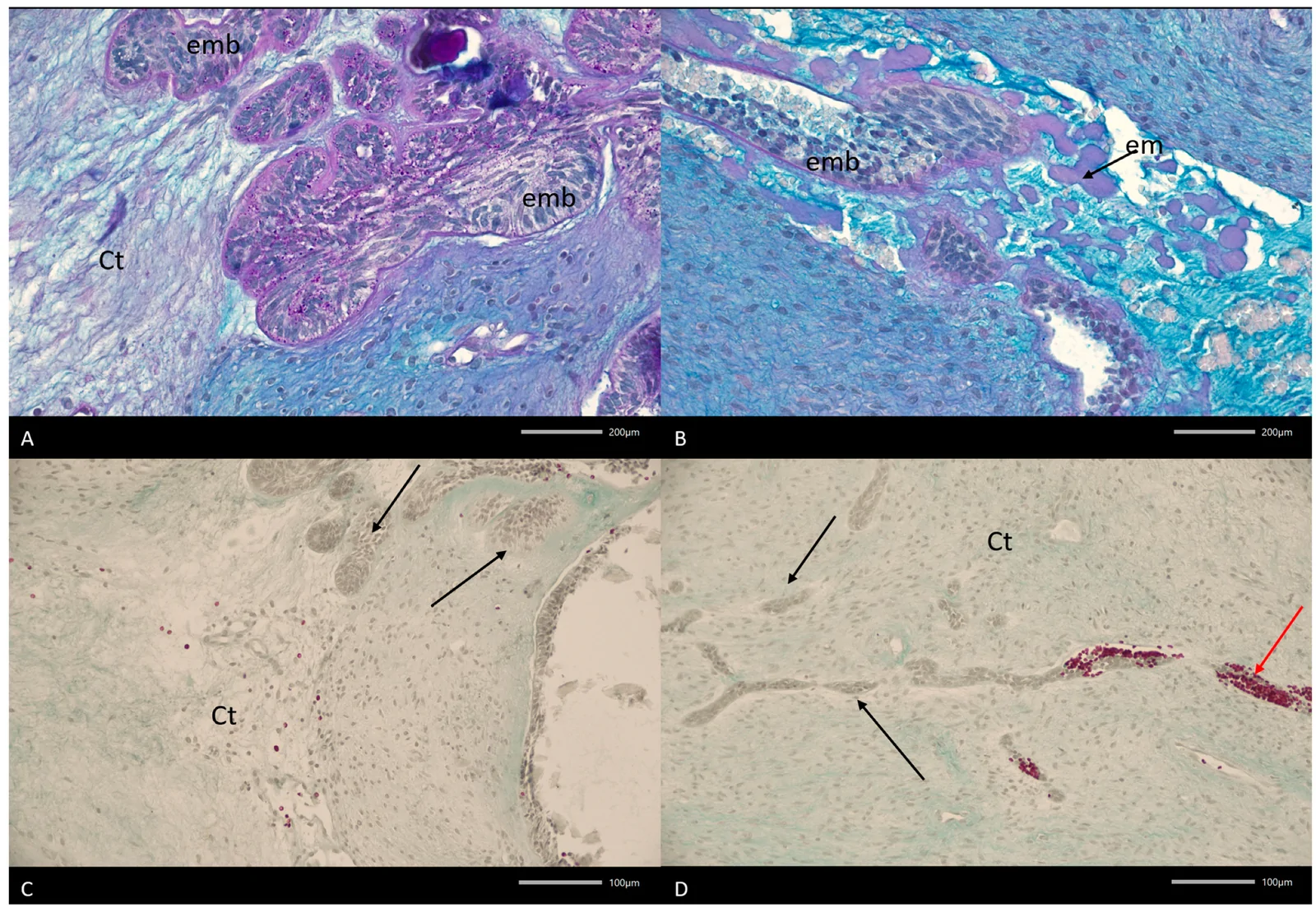
Within the specimen, numerous islands and strands of odontogenic epithelium are observed within the connective tissue (Ct), clearly demarcated from the surrounding stroma and separated by a distinct basement membrane, highlighted by PAS/Alcian Blue staining. The peripheral cells of the epithelial islands exhibit ameloblastic features (emb), appearing elongated with nuclei arranged in a palisading pattern and frequently demonstrating reversed polarity. Centrally, the epithelial islands contain cells with the morphology of stellate reticulum (**A**–**C**). The stroma is composed of loose, immature connective tissue of mesenchymal character, resembling dental papilla. Fibroblasts and a rich ground substance are observed. Small blood vessels accompany the epithelial strands (**D**). In the adjacent tissues, non-mineralized structures resembling enamel are present (**B**), whereas structures similar to other hard dental tissues are absent. (**A**)—Odontogenic epithelial islands and strands (emb) are visible within connective tissue (Ct), clearly separated from the stroma by a distinct basement membrane. (**B**)—Peripheral cells show ameloblastic features with palisading nuclei and reversed polarity, while central areas contain stellate reticulum–like cells. Adjacent tissues reveal non-mineralized enamel-like (em) structures without evidence of other hard dental tissues (**C**). The stroma consists of loose, immature mesenchymal tissue resembling dental papilla, with fibroblasts and abundant ground substance, and contains islets of enameloblastic epithelium (black arrow). (**D**)—Small blood vessels accompany epithelial strands (red arrow). (**A**,**B**)—PAS/Alcian Blue. (**C**,**D**)—Mallory trichrome. Mag. (**A**,**B**)—200× and (**C**,**D**)—100×. Numerous mitotic cells or any atypical mitotic figures, if noticed, might suggest a malignant entity in a very rare variant such as ameloblastic fibrosarcoma (AFS) [20,21,22,23,24,25,26,27,28].

**Figure 7:**
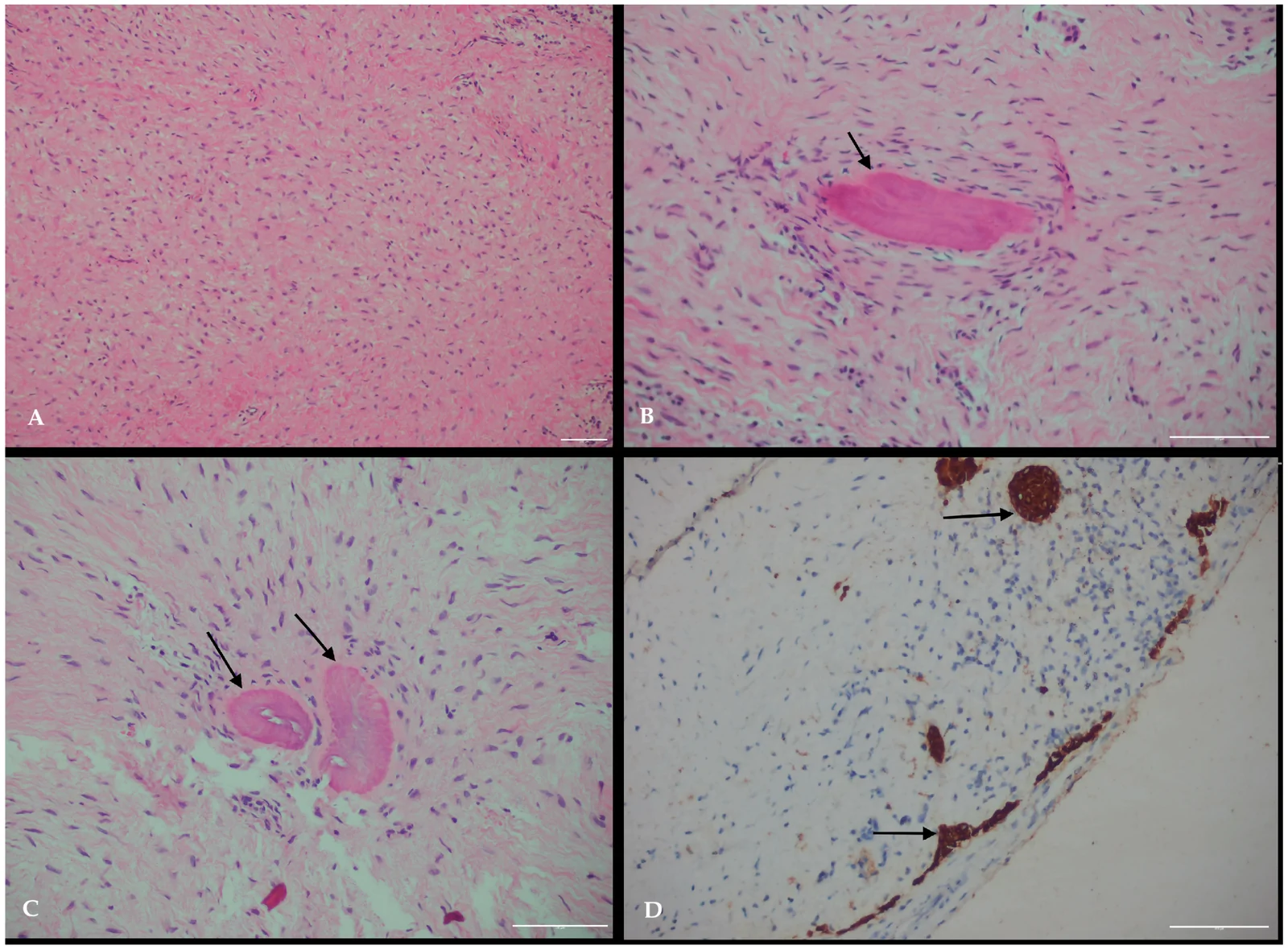
Representative histopathological images of a cystic-like lesion of the jaws diagnosed as odontogenic fibroma (scale bar: 100 µm, white). (**A**) Mesenchymal lesion composed of moderately cellular spindle cells without cytological atypia within a collagenous stroma (hematoxylin and eosin [H&E], ×100). (**B**,**C**) Focal mineralized, dentinoid-appearing, slightly basophilic cementum-like hard tissue formations are present within the lesion (arrows) (H&E, ×200). (**D**) Odontogenic epithelial remnants are observed as small, inactive islands and cords at the lesion periphery (arrows), showing pancytokeratin positivity on immunohistochemistry (×200). Histopathology remains the gold standard for every diagnosis; however, because of the growth of CT/CBCT, new technologies, like radiomics, imaging with the usage of AI, and neural links, improve the understanding of some bone lesion similarities and patterns. Some new systems with AI-improved differential diagnostics might bring some new insight and possibilities for better and faster diagnostics [29].

**Figure 8:**
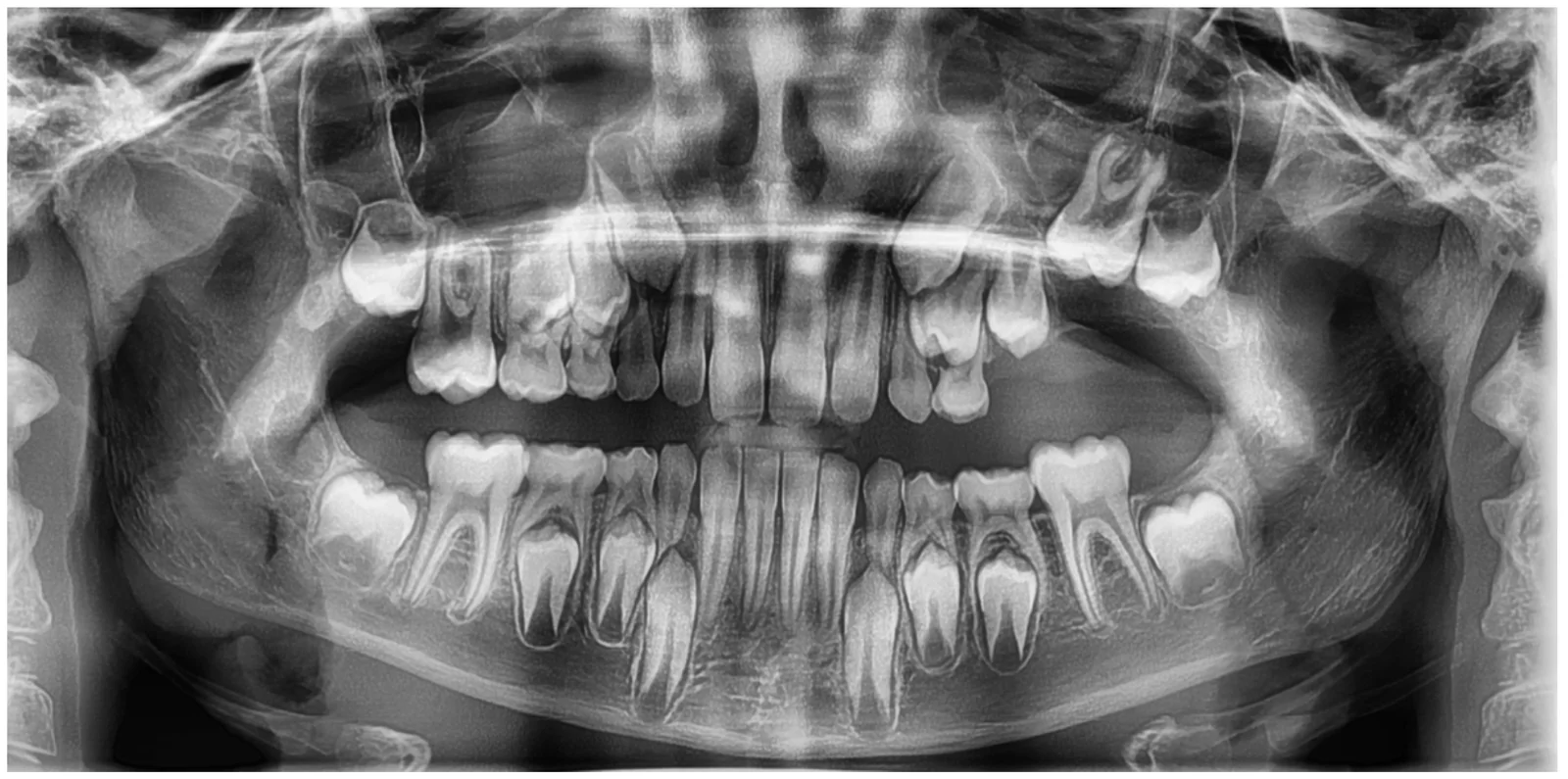
The long-term outcome in the AF case was favorable. Tooth eruption was not impaired, sinus-related symptoms improved, and normal bone growth was preserved. At the one-year follow-up, no evidence of tumor recurrence was observed. Bone healing was satisfactory, tooth eruption was not delayed, and the patient continued routine dental care. These radiographs demonstrate the favorable impact of early surgical intervention on subsequent patient outcomes. Owing to mixed dentition and the radiographic similarity of certain unilocular radiolucent lesions, each case requires an individualized and carefully planned diagnostic and therapeutic approach. In selected reports, adjunctive techniques such as Bichat’s fat pad transposition, bone grafting, local flaps, and prosthetic or orthodontic devices, including obturators, have been employed to promote healing and optimize wound care [20,21]. In selected cases, prosthetic obturators or mini-appliances may be employed to decompress advanced cystic lesions. However, optimal management requires individualized decision-making supported by thorough clinical evaluation and CBCT-based radiological assessment. Both cases demonstrate that accurate diagnosis requires CBCT in combination with FNAB, histopathology, and careful clinical examination of tooth-bearing maxillary structures, an approach that directly guided early surgical management. In each case, histopathological analysis established the definitive lesion type and confirmed the odontogenic tumor diagnosis. For more aggressive tumors, a radical treatment approach may be indicated [18,19,20,21,22,23,24]. These cases underline that the correct diagnostic workflow for pediatric unilocular radiolucency is imaging first, FNAB second, and histopathology as the final determinant. In most situations, juvenile patients require careful monitoring in the coming years and evaluation of bone shape, teeth position, and adequate occlusion. If odontogenic tumor recurrence occurs, an individualized treatment approach is required. Diagnostic uncertainty may arise in cases involving atypical bone lesions or radiolucencies with unclear local status. In such situations, either a conservative wait-and-see approach or revision surgery may be appropriate; however, the final decision depends on lesion shape, size, location, and the extent of bone loss [24,25,26,27,28]. Loss of typical cystic characteristics on needle aspiration should prompt additional diagnostic workup and tailored surgical planning. Goyal et al.’s study on aspiration cytology in jaw osseous lesions concluded that it was 97.3% accurate, with 100% specificity, while Mukherjee et al. support the authors’ initial argument that each case should be individually evaluated for at least two combined diagnostic approaches [30,31]. Authors recommend a detailed follow-up, frequent for pediatric patients to monitor for potential recurrence—in the first three years, every three months, then every six months until the age of eighteen—including a clinical examination for bone asymmetry, tooth mobility, or any atypical swelling. When AF/OF are found, once a year, a CBCT with limited dosage to a specific anatomical area can provide more information than a normal routine OPG. After each surgery, a local dentist should examine teeth vitality at least two times every six months to ensure good teeth formation, their eruption, and the absence of periapical lesions, caries, and local caries-related factors.

## Data Availability

The original contributions presented in this study are included in the article. Further inquiries can be directed to the corresponding author.
