# Correction: Nelke et al. “Dry Tap” Fine-Needle Aspiration Biopsy as a Diagnostic Clue in Cyst-like Juvenile Jaw Lesions Mimicking Dentigerous Cysts on Panoramic Radiography and Cone-Beam Computed Tomography. *Diagnostics* 2026, *16*, 439

**DOI:** 10.3390/diagnostics16213508

**Published:** 2026-10-27

**Authors:** Kamil Nelke, Klaudiusz Łuczak, Ömer Uranbey, Büşra Ekinci, Angela Rosa Caso, Michał Gontarz, Maciej Janeczek, Zygmunt Stopa, Piotr Kuropka, Maciej Dobrzyński

**Affiliations:** 1 Maxillo-Facial Surgery Ward, EMC Hospital, Pilczycka 144, 54-144 Wrocław, Poland; 2 Academy of Applied Sciences, Health Department, Academy of Silesius in Wałbrzych, Zamkowa 4, 58-300 Wałbrzych, Poland; 3 Department of Oral and Maxillofacial Surgery, Faculty of Dentistry, Aydın Adnan Menderes University, 09010 Aydın, Türkiye; 4 Department of Medical Pathology, Faculty of Medicine, Aydın Adnan Menderes University, 09010 Aydın, Türkiye; 5 Department of Oral and Maxillo-Facial Surgery, University of Siena, Viale Aldo Moro, 2, 53100 Siena, SI, Italy; 6 Department of Cranio-Maxillofacial Surgery, Jagiellonian University Medical College, Jakubowskiego 2 Street, 30-688 Cracow, Poland; michal.gontarz@uj.edu.pl; 7 Department of Biostructure and Animal Physiology, Wrocław University of Environmental and Life Sciences, Kożuchowska 1, 51-631 Wrocław, Poland; 8 Department of Cranio-Maxillofacial Surgery, Oral Surgery and Implantology, Medical University of Warsaw, Lindleya 4, 02-005 Warsaw, Poland; 9 Division of Histology and Embryology, Department of Biostructure and Animal Physiology, Wrocław University of Environmental and Life Sciences, Cypriana K. Norwida 25, 50-375 Wrocław, Poland; 10 Department of Pediatric Dentistry and Preclinical Dentistry, Wrocław Medical University, Krakowska 26, 50-425 Wrocław, Poland

In the published publication [1], there was an error regarding the affiliation for Michał Gontarz. The authors has requested that Michał Gontarz’s affiliation should be changed to ^6^ Department of Cranio-Maxillofacial Surgery, Jagiellonian University Medical College, Jakubowskiego 2 Street, 30-688 Cracow, Poland; michal.gontarz@uj.edu.pl. The authors state that the scientific conclusions are unaffected. This correction was approved by the Academic Editor. The original publication has also been updated.
